# MicroRNA in Acupuncture Studies: Does Small RNA Shed Light on the Biological Mechanism of Acupuncture?

**DOI:** 10.1155/2019/3051472

**Published:** 2019-04-16

**Authors:** Jade Heejae Ko, Seung-Nam Kim

**Affiliations:** ^1^College of Korean Medicine, Dongguk University, Goyang 10326, Republic of Korea; ^2^Graduate School, Dongguk University, Seoul 04620, Republic of Korea

## Abstract

MicroRNAs (miRNAs) are the main regulators of diverse physiological processes. Recently, miRNAs have emerged as significant players related to the effect of acupuncture although the biological mechanisms connecting the function of these miRNAs with the effect of acupuncture are not well understood. In animal models of various diseases, such as neurological disease, cardiovascular disease, myopathy, and pain, a number of miRNAs were altered after administration of electroacupuncture or manual acupuncture. Nonetheless, there are a limited number of studies published so far. This paper reviewed and discussed whether miRNAs could elucidate potential biological mechanism of acupuncture in the future studies.

## 1. Introduction

MicroRNA (miRNA) is a small molecule containing about 23 noncoding nucleotides. miRNA was first discovered in* Caenorhabditis elegans* and it was known by its function of repressing* lin-14* expression [1], and additional roles of miRNAs were found in many physiological processes, such as development [2–4], cell death process [5], and cell signaling [6]. Besides, emerging studies have shown that miRNAs contribute to the pathogenesis of neurological disease [7–9], cardiovascular disease [10, 11], metabolic syndrome [12–14], and cancer [15–17]. Acupuncture treatment, a frequently used therapeutic method in East-Asian medicine, is performed by penetrating specific point on skin, as known as acupuncture point (acupoint), with a filamentous needle. Two types of acupuncture treatment, manual acupuncture (MA), and electroacupuncture (EA) are distinguished by its treatment method. MA is performed with needle manipulation by adjusting insertion depth, rotation, and insertion duration, whereas EA involves running electric current through needles. There are a number of acupuncture studies showing its clinical effects on neuromuscular system [18–20], opioid system [21–24], immune regulation [25, 26], hormone regulation [27, 28], and so on. Interestingly, growing evidences in animal studies have shown that miRNAs may be a possible mean to explain underlying effect of acupuncture although a possible mechanism connecting the function of these miRNAs with the effect of acupuncture has not been studied enough. Here, we reviewed recent publications related to the acupuncture and its biological mechanism via miRNA expression.

## 2. Main Text

### 2.1. Stroke and Cardiovascular Disorder

The efficacy of electroacupuncture (EA) was demonstrated by enhancing blood flow and protecting nerve injury as summarized in Table 1. Deng* et al*. made an inquiry about how EA could possibly enhance rehabilitation after ischemic stroke and* miR-181b* was specifically studied as a mediator in the rehabilitation process. EA was administered on the acupoint GV20 of focal cerebral ischemic rat model and* miR-181b* expression was remarkably downregulated in the penumbra [29]. Chen* et al*. also showed that acupuncture at acupoints ST36 and GV20 potentially alter serum expression level of* miR-124* in rats with cerebral ischemia-reperfusion injury. It was shown that the lesion was relieved by acupuncture at the two acupoints, and* miR-124* expression was increased within the acupuncture group compared with model group [30]. Zheng* et al*. evaluated how EA ameliorates cerebral blood flow and neurological deficit after stroke. The middle cerebral artery occlusion (MCAO) rat model was used and EA was administered on acupoints GV26 and PC6. After administration of EA, cerebral blood flow was increased, and neurological function was improved as well. In this study,* miR-494* was downregulated and* miR-206* was upregulated in the penumbra [31]. Zhou* et al*. published a study in which a bioinformatic analysis of 20 miRNAs was used to test underlying neuroprotective mechanism of EA for ischemic injury. MCAO-induced focal cerebral ischemia rat models were used and rats received EA treatment daily on the acupoint GV20. Zhou et al. reported that downregulation of* miR-191a* and upregulation of neuronal calcium sensor 1 may result in an alleviation of neuron injury and consequently neuroprotection effect [32]. Liu* et al*. also investigated neuroprotective mechanism of nuclear factor kappa-light-chain-enhancer of activated B cells signaling pathway by EA on acupoints LI11 and ST36, and* miR-9* may be involved in the process of protection. According to their result, expression level of* miR-9* was significantly increased in the penumbra after EA treatment [33]. Zheng* et al*. studied efficacy of EA on learning and memory deficit, yet 2-vessel occlusion rat model was used for the study. EA was done on the acupoints GV14 and GV20. Compared with pre-EA treatment condition, reduction of* miR-132* was deferred after EA treatment [34].

The changes of miRNA by acupuncture in the cardiovascular-related diseases are summarized in Table 1. Wang* et al*. tested whether adenosine could mimic the effects of acupuncture via screening miRNAs. Spontaneously hypertensive rats were used and acupuncture at acupoint LR3 was given to each rat. The result confirmed that adenosine stimulation mimicked the effects of acupuncture and* miR-339, miR-223, miR-145* expressions were upregulated in the brain [35]. Acupuncture treatment at nonacupoint was also performed within the same group, and the result showed that increased expression of the miRNAs by acupuncture at acupoint LR3 had not occurred at nonacupoint [36]. Another protective role of miRNA is addressed and studied in myocardial ischemia/reperfusion model. Liu* et al*. explored a possible cardioprotective mechanism of EA via screening* miR-214*. The EA was done on acupoint PC6. It was revealed that the expression of* miR-214* was upregulated in the region of myocardial infarction. The study suggested that* miR-214* contributes to protective mechanism of EA [37].

### 2.2. Neurological Disorder

Table 2 shows the effect of acupuncture on changes of miRNA in neurological disorder animal model. Zhu* et al*. studied potential mechanism of EA on spinal cord injury (SCI) via* miR-449a*. They performed EA on acupoints GV6 and GV9 of SCI rat model and found that the expression of* miRNA-449a *was downregulated in the spinal cord by EA [38]. Liu* et al*. also studied responsiveness of EA on SCI rat model. Acupoints GV6 and GV9 were used for the EA and the result showed that EA improved functional recovery as* miR-214 *hindered neuronal apoptosis [39]. Su* et al*. examined how low frequency electrical stimulation (LFES) could improve muscle wasting which is caused by diabetes. Diabetes model was used and LFES was administered on two acupoints: GB34 and ST36. The result indicated that* miR-133a* is significantly increased in the skeletal muscle of diabetic mice group and muscle weight was higher in diabetic mice which LFES was given to [40].

Moreover, there are studies reporting how EA could enhance cognitive and mood disorder as shown in Table 2. First, Liu* et al*. studied* miR-134* in the rat hippocampus with middle cerebral artery occlusion induced cognitive deficit (MICD). The EA treatment was done on acupoints GV20 and GV24. The expression of* miR-134* was increased in MICD group compared with normal group, whereas expression of* miR-134* was decreased after repeated administration of EA [41]. EA responsiveness on depression was studied by Duan* et al*. EA was administered on acupoints GV20 and EX-HN3 of depressive rat model. After the EA,* miR-383* and* miR-764* were decreased and* miR-1* was increased in the cerebral cortex compared with model group [42].

Su* et al*. published another study that evaluated whether acupuncture with LFES would improve denervation-induced muscle atrophy (Table 2). The LFES treatment was done on acupoint GB34 and ST36 of denervation-induced mice model. The results demonstrated that LFES stimulated expression of* miR-1* and* miR-206* as its expression was increased in the skeletal muscle [43].

Effect of acupuncture treatment on insomnia was studied by Bo* et al*. (Table 2). They used parachlorophenylalanine-induced insomnia rat models and performed Mongolian medial warm acupuncture treatment. After the treatment, they detected upregulation of* miR-101a *in the brain and suggested that the upregulation of* miR-101a* is probably related to paired box gene 8 regulation [44].

### 2.3. Other Disease Animal Models

Unlike previous studies which are using disease model, Cui* et al*. studied EA tolerance in rat (Table 3). Given that EA tolerance may affect responsiveness of EA as it is continued for a long period of time, Cui* et al*. screened miRNAs in order to test whether the expression levels are altered. After the EA administration on acupoints ST36 and SP6 for 8 consecutive days, expression levels of* miRNA-124* and* miRNA-107* were increased, whereas expression levels of* miR-148* and* miR-370* were decreased in the hypothalamus compared with initial level [45].

Effect of MA in inflammatory diseases was also studied. Zhang* et al*. evaluated potential therapeutic mechanism of acupuncture in treatment of chronic atrophic gastritis via screening miRNAs (Table 3). A group of chronic atrophic gastritis rat models received acupuncture treatment on acupoints ST36, CV12, and BL20. As a result of the acupuncture treatment, the expression levels of* miR-155* and* miR-21* were downregulated, whereas the expression of* miR-339* was upregulated in the gastric tissue after the MA treatment [46].

A study of Deng* et al*. highlighted a possible involvement of miRNA in treatment of postoperative ileus by acupuncture. Postoperative ileus mouse model was used and acupuncture was administered on acupoints ST36, SP6, and LR3. Serum* miR-19a* level was noticeably increased after inducing postoperative ileus. Acupuncture treatment downregulated the increase and probably protected interstitial cells as deferring expression of* miR-19a* [47].

Hu* et al*. studied how LFES affects insulin-like growth factor 1 signaling pathway and how LFES alters miRNA expression which would potentially improve muscle protein synthesis, while impeding protein degradation (Table 3). For the study, chronic kidney disease-induced muscle atrophy mice model was used. Using qPCR assays, they detected a significant decrease of* miR-1* level in the skeletal muscle although its level gradually returned to the initial level [48].

Impact of EA on the regulation of hypothalamic hormone was studied by Zhu* et al*. (Table 3). Hyperactivity of the hypothalamus pituitary adrenal axis was induced via hepatectomy in rat model. EA treatment was performed on acupoints ST36 and SP6. They showed that expression levels of* miR-142* and* miR-376c* were both decreased in the hypothalamus after EA treatment [49].

Wang* et al*. investigated the effect of EA administration using allergic contact dermatitis rat model (Table 3). EA was administered on acupoint ST36 on rat and rat peritoneal mast cells were obtained for miRNA screening. It was found that* miR-155* level was downregulated by EA treatment in rat peritoneal mast cells.

## 3. Discussion

In this study, we reviewed various studies which have shown the effect of MA and EA in different disease models via miRNA profiling. Based on the results of above-mentioned studies, it was shown that expression levels of various miRNAs, which were thought to play significant roles in various diseases, have been changed by acupuncture treatment.

With regard to stroke and its link with miRNA, expression levels of* miR-124* and* miR-9 *were increased by acupuncture treatment in ischemic rat models [30].* miR-124* has been studied on its inhibitory role in neural apoptosis occurred by ischemic stroke [50]. Mediating role of* miR-9 *was suggested as expression level of* miR-9* was decreased in middle cerebral artery occlusion rat model; yet, neurological functions and behavioral abnormalities were restored with* miR-9* level restoration [51]. Also, increased level of* miR-132* influenced on adjusting angiogenesis by suppressing NF-*κ*B and vascular endothelial growth factor in patients with ischemic cerebrovascular disease [52]. These changes and distinct roles of each miRNAs may possibly provide an intriguing connection between the effect of acupuncture on stroke and these miRNAs.

There are other miRNAs potentially associated with cardiovascular disorders. By analyzing literatures, it was suggested that* miR-145*, which was increased by acupuncture in hypertension rat model [36], plays a significant role in different cardiovascular diseases [53]. Also, low blood pressure was evident in* miR-145* knockdown mice and the change of blood pressure was considered to be a result of reduced vascular tone which would possibly be controlled by* miR-145* [54, 55].* miR-214* was increased by acupuncture treatment in myocardial ischemia-reperfusion rat model [39], and local upregulation of* miR-214* in heart alleviated cardiac dysfunctions in dilated cardiomyopathy patients [56]. Moreover, in a diabetic cardiac dysfunction study,* miR-1* level was restored by N-acetylcysteine treatment in diabetic rat model [57].

Nerve injury studies also identified candidate miRNAs associated with acupuncture treatment.* miR-449*, which was changed by acupuncture in nerve injury model [38], was downregulated as oligodendrocyte precursor cell was transplanted to spinal cord injury rat model. Moreover,* miR-206* promoted regeneration of neuromuscular synapses in ALS model [58] and brain-derived neurotrophic factor-related signaling pathway in nerve injured neuropathic pain model [59]. Therefore, it was suggested that* miR-206* and* miR-449a* might be associated with potential treatment method of nerve injury.

It was suggested that dysregulation of miRNAs is possibly associated with physical dysfunctions and abnormalities. Here, based on analysis of the published literatures, we summarized that acupuncture treatment seemingly restores levels of diverse miRNAs, which were thought to play significant roles in various diseases, to the normal states, suggesting that these miRNAs potentially have influential roles in the effect of acupuncture on these diseases. Although it should be critically considered that there are methodological and hypothetical differences between the studies, the associations are intriguing and worthy to be analyzed and studied further.

There are still relatively limited number of studies about acupuncture and miRNAs. Nevertheless, this manuscript highlighted the effect of EA and MA and in what way miRNAs have taken part in elucidating mechanism of acupuncture. New findings could conceivably lead to discovering biological mechanism of acupuncture via miRNA and it would shed light on identification of underlying mechanism of acupuncture in the near future.

## Figures and Tables

**Table 1:** Changes of microRNAs by acupuncture treatment in the stroke and cardiovascular disorder animal model.

Disease model	Author	Year	Species	EA/MA	Acupoint	Acupuncture parameters	Tissue	Result
Stroke	Deng *et al.*	2016	Rat	EA	GV20	2 Hz/10 Hz, 1 mA, 30 min	Brain	*↑ miR-181b*
	Zheng *et al.*	2016	Rat	EA	GV20, GV14	20 Hz, 20 min	Brain (cortex)	*↑ miR-132*
	Zheng *et al.*	2016	Rat	EA	GV26, PC6	2 Hz, 0.3 mA, 1 min	Brain	*↑ miR-206, ↓ miR-494*
	Liu *et al.*	2016	Rat	EA	LI11, ST36	1 Hz/20 Hz, 30 min	Brain	*↑ miR-9*
	Zhou *et al.*	2017	Rat	EA	GV20	1 mA	Brain	*↓ miR-191a*
	Chen *et al.*	2016	Rat	EA	GV20, left ST36	2 Hz, 1 mA, 20 min	Heart, blood	*↑ miR-124*

Cardiovascular disorder	Wang *et al*.	2014	Rat	MA	LR3	3 mm depth, 5 min	Brain (medulla)	*↑ miR-339, miR-145, miR-451*
	Liu *et al.*	2014	Rat	EA	PC6	4 Hz/20 Hz, 0.5 ms, 1 mA, 30 min	Heart	*↑ miR-214*
	Wang *et al.*	2015	Rat	MA	LR3	N/A	Brain (medulla)	*↑ miR-339, miR-223, miR-145*

EA; electroacupuncture, MA; manual acupuncture.

**Table 2:** Changes of microRNAs by acupuncture treatment in the neurological disorder animal model.

Disease model	Author	Year	Species	EA/MA	Acupoint	Acupuncture Parameters	Tissue	Result
Nerve injury	Zhu *et al.*	2017	Rat	EA	GV6, GV9	1 mA	Spinal cord	*↓ miR-449a*
	Su *et al.*	2016	Mouse	EA	GB34, ST36	20 Hz, 1 mA, 15 min	Muscle	*↑ miR-1, miR-206*
	Liu *et al.*	2017	Rat	EA	GV6, GV9	60 Hz, ≤ 1 mA, 20 min	Brain (hippocampus)	*↑ miR-214*

Cognitive and Mood disorder	Liu *et al.*	2017	Rat	EA	GV20, GV24	1 Hz/20 Hz, 0.2 mA, 30 min	Brain	*↓ miR-134*
	Duan *et al.*	2016	Rat	EA	EX-HN3, GV20	2 Hz, 1 mA, 20 min	Blood serum	*↑ miR-1, ↓ miR-383, miR-764*

Denervation-induced muscle atrophy	Su *et al.*	2015	Mouse	EA	GB34, ST36	20 Hz, 1 mA, 15 min	Muscle	*↑ miR-1, miR-206, miR-133a, miR-133b*

Insomnia	Bo *et al.*	2017	Rat	MA	EX-HN3, GV9, GV14	15 min	Brain (prefrontal cortex, hypothalamus, and hippocampus)	*↑ miR-101a*

EA; electroacupuncture, MA; manual acupuncture.

**Table 3:** Changes of microRNAs by acupuncture treatment in the other disease animal models.

Disease model	Author	Year	Species	EA/MA	Acupoint	Acupuncture parameters	Tissue	Result
EA tolerance	Cui *et al.*	2017	Rat	EA	ST36, SP6	2 Hz/15 Hz,	Brain (hypothalamus)	*↑ miR-124, miR-107,*
						3mA, 30 min		*↓ miR-148a, miR-370*
Postoperative ileus	Deng *et al*.	2017	Rat	MA	ST36, SP6,	2-3 mm depth,	Colon	*↓ miR-19a*
					LR3	15 min		
Chronic atrophic gastritis	Zhang *et al*.	2016	Rat	MA	ST36, CV20,	3 mm depth,	Gastric tissue	*↑ miR-339,*
					BL20	15 min		*↓ miR-155, miR-21*
Chronic kidney disease	Hu *et al.*	2015	Mouse	EA	GB34, ST36	20 Hz, 1 mA,	Muscle	*↓ miR-1*
						15 min		
Hyperactivity of HPA axis	Zhu* et al.*	2017	Rat	EA	ST36, SP6	2 Hz/15 Hz,	Brain (hypothalamus)	*↑ miR-142, miR-376c*
						2mA, 30 min		
Allergic contact dermatitis	Wang et al.	2018	Rat	EA	ST36	2 Hz, 1 mA,	Ear	*↓ miR-155*
						5 min, 20 min		

EA; electroacupuncture, MA; manual acupuncture, HPA; hypothalamic-pituitary-adrenal.
